# Chlorhexidine vs. Povidone for Skin Antisepsis in Tissue Expander-Based Breast Reconstruction: A Propensity Score-Matched Analysis

**DOI:** 10.3390/jcm14165734

**Published:** 2025-08-13

**Authors:** Agustin N. Posso, Audrey Mustoe, Manuela Neira, Micaela Tobin, Mohammed Yamin, Tricia Raquepo, Maria J. Escobar-Domingo, Sarah J. Karinja, Bernard T. Lee

**Affiliations:** Division of Plastic and Reconstructive Surgery, Beth Israel Deaconess Medical Center, Harvard Medical School, Boston, MA 02215, USA; aposso@bidmc.harvard.edu (A.N.P.); amustoe@luc.edu (A.M.); mneira@bidmc.harvard.edu (M.N.); micaela.tobin1@umassmed.edu (M.T.); myamin1@bidmc.harvard.edu (M.Y.); traquepo@bidmc.harvard.edu (T.R.); mjescobardomingo@hsph.harvard.edu (M.J.E.-D.); skrinja@bidmc.harvard.edu (S.J.K.)

**Keywords:** breast reconstruction, tissue expander, chlorhexidine, povidone, surgical site infection

## Abstract

**Background/Objectives**: Tissue expander (TE)-based breast reconstruction is a common procedure, but postoperative infection rates can reach up to 30%. The optimal skin antiseptic solution for minimizing these infections remains uncertain. This retrospective cohort study aimed to compare the impact of chlorhexidine and povidone-iodine for skin antisepsis in preventing surgical site infections in patients who underwent TE-based breast reconstruction. **Methods**: The TriNetX database was queried to identify patients who underwent TE-based breast reconstruction. Patients were classified into two cohorts: the chlorhexidine group and the povidone-iodine group. A propensity score matching analysis was performed to control infection risk factors. The primary outcome was the occurrence of surgical site infections, while secondary outcomes included wound dehiscence, emergency department visits, debridement, and TE removal. All outcomes were assessed at 30, 60, and 90 days following surgery. **Results**: After matching of both the chlorhexidine cohort and povidone-iodine cohort, each consisted of 1446 patients. Within 30 days post-surgery, no significant differences were observed between the chlorhexidine and povidone-iodine groups in terms of the risk of surgical site infections (RR 0.62, *p* = 0.168), wound dehiscence (RR 1.00, *p* = 1.000), emergency department visits (RR 0.95, *p* = 0.700), debridement (RR 0.71, *p* = 0.271), or TE removal (RR 0.84, *p* = 0.335). Similar results were seen at 60 and 90 days post-surgery. **Conclusions**: This study suggests that chlorhexidine and povidone-iodine may be equally effective for skin antisepsis in preventing surgical site infections and associated complications in patients undergoing TE-based breast reconstruction.

## 1. Introduction

Tissue expanders (TEs) are commonly used in breast reconstruction, with approximately 70,000 procedures performed annually in the United States [1]. However, surgical site infections (SSIs) following these procedures remain a significant concern, with infection rates reported to be as high as 30% [2]. SSIs can lead to increased reoperation rates, prolonged hospital stays, and higher healthcare costs [1]. Various surgical risk factors, such as prolonged procedures and inadequacies in the surgical scrub or antiseptic skin preparation, may contribute to the risk of infection [3]. As a result, effective patient skin antisepsis, based on evidence, is critical to enhancing patient safety and minimizing the risk of SSIs [4].

Among the most commonly used antiseptic solutions for skin preparation are chlorhexidine and povidone-iodine [5]. Chlorhexidine works by a cationic attraction to the bacterial cell wall’s negatively charged phosphate components, which damages the cytoplasmatic membrane, causing ion leakage and inhibiting membrane enzymes [6]. It also provides broad-spectrum antimicrobial coverage, rapid bactericidal activity, prolonged residual effects, and minimal irritation [7,8]. On the other hand, povidone-iodine rapidly penetrates bacterial cells and oxidizes proteins, nucleotides, and fatty acids, resulting in cell death [9]. Povidone-iodine is known for its broad antimicrobial spectrum, lack of resistance, effectiveness against biofilms, and good tolerability [10].

Although numerous studies have compared chlorhexidine and povidone-iodine for skin antisepsis in various settings, the evidence remains conflicting. For example, some studies have shown a superior efficacy of chlorhexidine in preventing infections after catheter insertion [11] or clean-contaminated surgery [12]. Conversely, other research in thoracic and abdominal surgery has found no significant differences in infection rates between these two antiseptics [13]. However, no studies have specifically compared the effectiveness of chlorhexidine and povidone-iodine in preventing SSIs following TE-based breast reconstruction. This retrospective cohort study aims to fill that gap by evaluating the impact of chlorhexidine versus povidone-iodine as skin antiseptics on SSI rates in patients undergoing TE-based breast reconstruction using a large real-world database (TriNetX).

## 2. Materials and Methods

This retrospective cohort study aimed to assess the risk of SSIs at 30, 60, and 90 days in patients who underwent TE-based breast reconstruction. The study compared chlorhexidine and povidone for skin antisepsis, using data from the TriNetX database. TriNetX (Cambridge, MA, USA) aggregates deidentified records from 97 healthcare organizations, covering more than 130 million patients. It provides real-time access to a wide range of electronic health data, including diagnosis, procedures, medications, laboratory results, and genomic data. As an important note, if the number of patients in a particular group is less than 10, TriNetX will round up and display the number as 10 to protect patient privacy. This study was reviewed and granted an exemption by the Beth Israel Deaconess Medical Center Institutional Review Board (ID 2024D000940). Data extraction relied on Current Procedural Terminology (CPT), International Classification of Diseases, Revision 10 (ICD-10), and RxNorm codes, which are detailed in Table A1.

### 2.1. Cohorts

**Figure 1:**
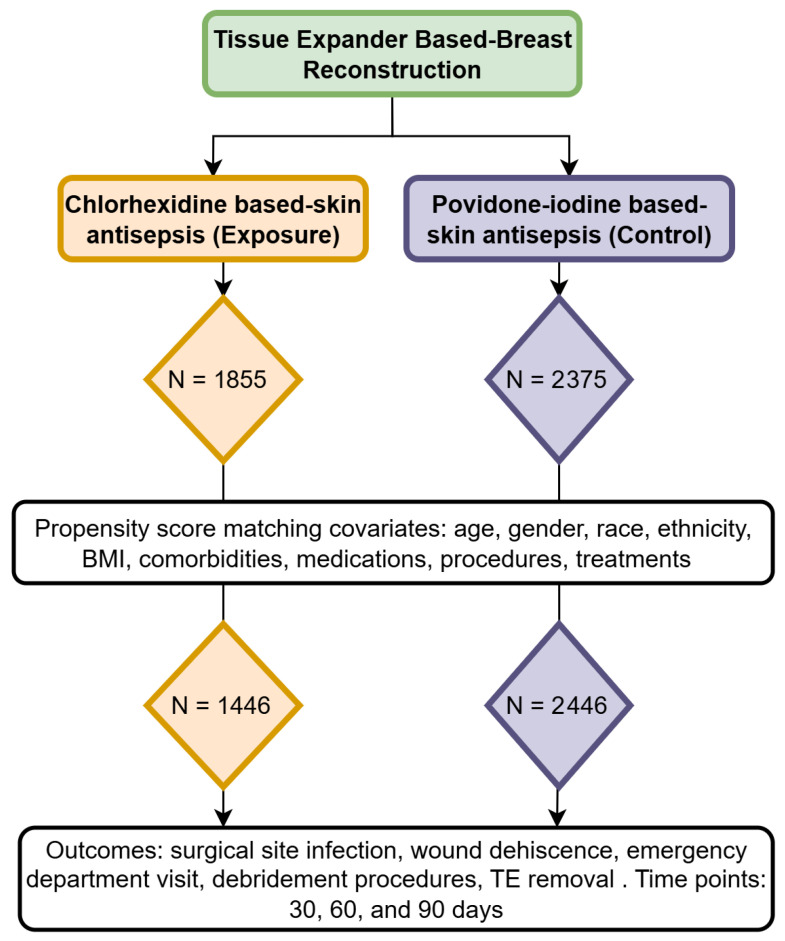
Flowchart of cohort selection and propensity score matching for tissue expander-based breast reconstruction.

The chlorhexidine cohort consisted of patients who underwent TE-based breast reconstruction and for whom chlorhexidine was used for skin antisepsis; this group was designated as the exposed cohort. The povidone cohort included patients who also underwent TE-based breast reconstruction but for whom skin antisepsis was performed with povidone-iodine; this group was designated as the control cohort (Figure 1).

### 2.2. Outcomes

All outcomes were assessed at 30, 60, and 90 days postoperatively. The primary outcome was SSI, and secondary outcomes included wound dehiscence, emergency department visits, need for debridement, and TE removal.

### 2.3. Statistical Analysis

All statistical analyses were completed on the TriNeTx online research platform. Baseline characteristics were compared with chi-squared tests for categorical variables and independent *t* tests for continuous variables. The TriNetX platform was used to conduct a 1:1 propensity score matching with logistic regression. The matching employed nearest-neighbor matching with a caliper of 0.1, ensuring that the standardized mean differences between propensity scores were ≤0.1 post-matching [14]. This threshold was selected to maximize baseline covariate balance between cohorts and minimize residual confounding, as calipers wider than 0.1 have been shown to compromise the quality of matching and increase bias [15]. The following covariates were used in the propensity score matching: demographics (age, sex, race, ethnicity), body mass index, comorbidities (tobacco use, diabetes, hyperlipidemia, hypertensive diseases, immune disorders, acute and chronic kidney disease, breast cancer), procedures (breast biopsy, biologic implant placement), medication use (immunosuppressants, corticosteroids), and other treatments (radiation, chemotherapy). For all outcomes, risks, risk ratios (RRs), and 95% confidence intervals (CIs) were assessed. A *p*-value < 0.05 was considered statistically significant. Power analysis was conducted using R Studio (Version 4.4.0) and the “pwr” package for chi-square tests. Based on SSI rates from a previous similar study [16]—14.6% in the chlorhexidine group and 4.5% in the povidone-iodine group—and our total sample size of 2982 patients, the analysis, using a significance level of 0.05, yielded a statistical power greater than 0.80.

## 3. Results

This study initially assessed 1855 patients in the chlorhexidine cohort (mean age of 51.6 years, SD 11.2) and 2375 patients in the povidone-iodine cohort (mean age 50.6 years, SD 11.5) (*p* = 0.007). Significant differences between the cohorts were observed across several characteristics, as detailed in Table 1. After matching, each cohort included 1446 patients, with no significant differences for all covariates (Table 2). For the 30-, 60-, and 90-day outcomes, the mean follow-up times (±SD) in the exposure cohort were 29.65 ± 2.80, 58.80 ± 7.11, and 87.35 ± 12.59 days, respectively. In the control cohort, the corresponding mean follow-up times were 29.73 ± 2.50, 58.93 ± 6.53, and 87.48 ± 11.92 days, respectively.

**Figure 2:**
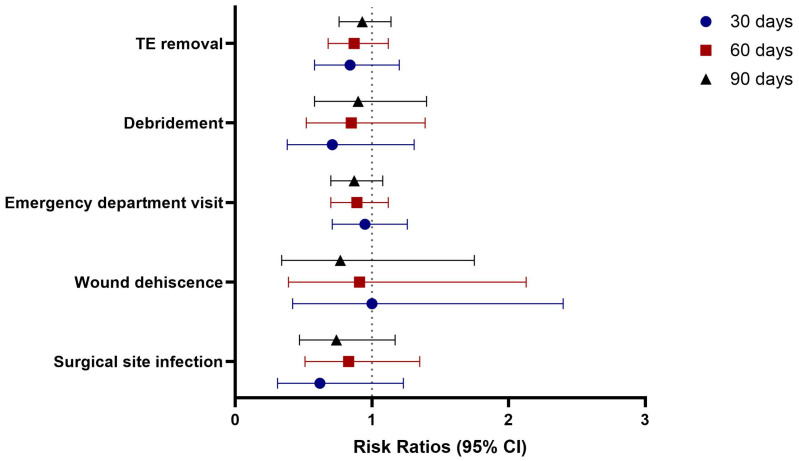
Comparison of outcomes in patients who underwent TE-based breast reconstruction: chlorhexidine vs. povidone-iodine skin antisepsis.

At 30 days post-surgery, the chlorhexidine cohort showed non-significant differences in the risks of surgical site infections (RR 0.62, *p* = 0.168), wound dehiscence (RR 1.00, *p* = 1.000), emergency department visits (RR 0.95, *p* = 0.700), debridement (RR 0.71, *p* = 0.271), and TE removal (RR 0.84, *p* = 0.335) compared to the povidone-iodine cohort (Table 3, Figure 2).

At 60 days post-surgery, outcomes remained comparable between the two cohorts, with no significant differences in surgical site infections (RR 0.83, *p* = 0.448), wound dehiscence (RR 0.91, *p* = 0.827), emergency department visits (RR 0.89, *p* = 0.320), debridement (RR 0.85, *p* = 0.524), or TE removal (RR 0.87, *p* = 0.275) (Table 3, Figure 2).

Similarly, at 90 days post-surgery, the chlorhexidine cohort maintained non-significant differences in the risks of surgical site infections (RR 0.74, *p* = 0.198), wound dehiscence (RR 0.77, *p* = 0.530), emergency department visits (RR 0.87, *p* = 0.197), debridement (RR 0.90, *p* = 0.642), and TE removal (RR 0.93, *p* = 0.478) compared to the povidone-iodine cohort (Table 3, Figure 2).

## 4. Discussion

This retrospective cohort study evaluated the risk of SSIs in patients who underwent TE-based breast reconstruction, comparing those who received chlorhexidine with those who received povidone-iodine for skin antisepsis. The results demonstrated no statistically significant differences in the risk of developing SSIs between the two cohorts at 30, 60, and 90 days post-surgery. Additionally, there were no significant differences in secondary outcomes, including wound dehiscence, emergency department visits, debridement, and TE removal. These findings suggest that chlorhexidine and povidone-iodine may be equally effective in preventing SSIs and related complications in patients undergoing TE-based breast reconstruction.

Our results are consistent with a number of prior studies across various surgical specialties that have reported no clear superiority of either antiseptic. Several large meta-analyses support this finding. For instance, a large meta-analysis on intravitreal injections, involving 453,340 eyes, found no statistically significant difference in endophthalmitis rates between the two antiseptics (OR 1.26, 95% CI 0.53–3.00), suggesting comparable efficacy in this ophthalmologic procedure [17]. Similarly, a comparative meta-analysis of 10 randomized clinical trials in abdominal surgery reported no significant difference in SSI risk between povidone-iodine–alcohol and chlorhexidine–alcohol (RR 1.20, 95% CI 0.94–1.54) [18]. Although the study was methodologically rigorous, it highlighted heterogeneity among the included trials, which may have affected the generalizability of the study. In the orthopedic literature, another meta-analysis of 11 studies including 67,742 patients who underwent primary joint arthroplasty found no significant difference in joint infection rates between povidone-iodine and chlorhexidine (RR 1.60, *p* = 0.154) [19]. Despite the large sample size, the limitations included variations in surgical technique, which may also be a limitation of our study. Randomized controlled trials have yielded similar findings. A multicenter randomized controlled trial involving 5788 patients undergoing clean-contaminated, contaminated, or dirty abdominal surgery found no difference in SSI risk between alcohol-based chlorhexidine and povidone-iodine (RR 0.97, 95% CI 0.82–1.14) [20]. The pragmatic design, inclusion of low- and middle-income countries, and stratification between levels of contamination added external validity to this study. In a double-blind randomized clinical trial of 661 patients who underwent laparoscopic gynecologic surgery, the odds ratio for port-site infection with alcohol-based chlorhexidine compared to alcohol-based povidone-iodine was 1.34 (95% CI, 0.71–2.52) [21]. Notably, the study was adequately powered to detect a 10% difference in SSI rates between the two antiseptic agents. Additionally, a single-blinded, non-inferiority randomized clinical trial of 174 patients who underwent transsphenoidal surgery demonstrated no significant difference in postoperative nasal bacteria clearance between the chlorhexidine and povidone-iodine groups (88.64% vs. 82.56%, difference 6.10%; 95% CI −5.30 to 17.50). The authors concluded that both antiseptics were equally effective for nasal decolonization [22]. Retrospective studies, while well-designed, are inherently limited with regard to causality inference; however, they have also demonstrated comparable risks. For example, a retrospective analysis involving 592 patients who underwent breast, colon, and vascular surgeries found no difference in SSI rates between iodine–alcohol and chlorhexidine–alcohol groups (*p* = 0.20) [23].

On the other hand, other studies suggest that chlorhexidine may be more effective. A comprehensive meta-analysis with pooled data of clean-contaminated surgeries, including general and gynecologic procedures, found that chlorhexidine significantly reduced SSIs compared with povidone-iodine (OR 0.68, 95% CI 0.50–0.94) [24]. Another large meta-analysis involving 29,006 patients found chlorhexidine to be superior in preventing SSIs in clean and clean-contaminated surgeries (RR 0.65, *p* < 0.00001) [25]. While the large sample size lends strong statistical power to the findings, it is important to note that a subgroup analysis by surgical site was not conducted, which limits the applicability to specific procedures. Similarly, chlorhexidine was found to be more effective in reducing bacterial skin colonization compared to iodine (RR 0.45, 95% CI 0.36–0.55) [26]. This trend is supported by randomized controlled trials. For example, a randomized controlled trial involving 1000 patients undergoing peripheral venous catheter insertion found that local infections occurred less frequently with chlorhexidine–alcohol than with povidone–alcohol (0% vs. 1%). More importantly, catheter colonization was also reduced (1% vs. 17%), with a hazard ratio of 0.08 (95% CI 0.02–0.18) [27]. Similarly, a well-designed prospective cohort study of 2454 patients undergoing upper limb surgery found that chlorhexidine–alcohol significantly reduced SSI risk in elective surgeries compared with aqueous povidone-iodine (HR 0.30, 95% CI 0.11–0.84) [28]. Another retrospective study of 256 patients who underwent gynecological surgery reported that chlorhexidine–alcohol was more effective, reducing the overall rate of SSIs from 14.6% to 4.5% (*p* = 0.011) compared with the povidone-iodine group [16].

Interestingly, fewer studies have reported greater efficacy of povidone-iodine in preventing SSIs. A pediatric orthopedic study with 1416 cases and 1416 controls found in a subgroup analysis that chlorhexidine used in upper-extremity sports procedures was associated with 29 more infections per 1000 cases compared to povidone (*p* = 0.005) [29]. However, their propensity score matching model was limited by the sole inclusion of age and sex as covariates. In contrast our propensity matching model incorporated a more comprehensive set of variables, including body mass index, comorbidities, other procedures, and additional treatments, thereby improving the robustness of the comparison. Additionally, a study involving 16 patients undergoing shoulder surgery found that aerobic skin flora was more effectively reduced by alcohol-based povidone-iodine than by alcohol-based chlorhexidine (reduction factor 2.55 vs. 1.94, *p* = 0.04); similar results were found for coagulase-negative staphylococci and anaerobic flora [30].

Both chlorhexidine and povidone-iodine are broad-spectrum antiseptics with distinct mechanisms of action [31]. While chlorhexidine could be considered superior to povidone-iodine due to its rapid action, persistent activity despite exposure to body fluids, and long-lasting residual effect [12,32], our study—consistent with many others in various surgical settings—did not find that these pharmacological advantages result in a statistically meaningful reduction in SSIs following TE-based breast reconstruction.

It is possible that the inherently high infection risk associated with this procedure limits the impact of a single intervention in reducing SSIs. TE-based breast reconstruction involves the opening and manipulation of the breast ductal system [33], the potential compromise of blood supply after mastectomy [34], and the insertion of foreign material into the breast pocket [35], all of which contribute to the complexity of infection prevention. Therefore, relying on a single intervention may not be sufficient to reduce SSIs significantly.

Instead, implementing evidence-based bundled interventions may be necessary to effectively reduce infection risk in these patients. Such interventions could include adequate antibiotic prophylaxis, decolonization protocols, maintenance of perioperative normothermia, intraoperative wound lavage, blood glucose control, infection surveillance, staff education, patient and family education, and institutional policies supporting best practices [36,37,38,39,40,41]. Additionally, the role of different antiseptic techniques in preventing SSIs warrants further investigation in other underexplored areas of breast reconstruction, such as fat grafting [42].

## 5. Limitations

The authors of this study acknowledge several limitations. First, the use of the TriNetX database may have introduced selection bias, as it aggregates data from large healthcare organizations which may not fully represent the broader population, and this could limit the generalizability of the results. Second, the reliance on CPT, ICD-10, and RxNorm codes for defining cohorts and outcomes introduced the potential for inaccuracies or misclassification. Third, while the use of chlorhexidine or povidone-iodine was determined using an RxNorm code, the database did not provide details on concentrations or whether it was an alcoholic-based antiseptic. This limitation prevented a more granular analysis of the comparative effects of chlorhexidine and povidone-iodine on our outcomes. Fourth, although several covariates were included to balance the cohorts, unmeasured confounders—such as breast pocket irrigation solutions, operative time, postoperative care, inherited risk factors, or patient behaviors—may still have influenced the results. Finally, while there was a consistent trend towards a lower risk of SSIs and related complications in the chlorhexidine cohort across all time points, these differences did not reach statistical significance. This suggests the study could be underpowered, and larger sample sizes may be required to detect meaningful differences in future investigations.

## 6. Conclusions

In this large retrospective cohort study using TriNetX, we found no significant differences in the risk of SSIs or related complications between patients who underwent TE-based breast reconstruction with chlorhexidine or povidone-iodine for skin antisepsis. While both antiseptics are widely used, our findings suggest that, within this specific surgical context, the choice between these agents may not significantly impact the postoperative infection outcomes. These results may underscore the importance of evaluating antiseptic strategies in the context of broader perioperative infection bundles rather than relying solely on individual agents. Additionally, given the observational nature of this study, future prospective randomized trials are warranted to better control confounding and assess causality. Our findings also highlight the need for further research in underexplored areas of breast reconstruction such as fat grafting. Ultimately, SSI prevention should be approached through multifaceted interventions.

## Figures and Tables

**Table 1:** Chlorhexidine (*n* = 1855) and povidone-iodine (*n* = 2375) cohort characteristics before propensity score matching.

Covariate	Chlorhexidine Cohort (*n* = 1855)	Povidone Cohort (*n* = 1855)	*p*-Value
**Age**, mean (SD)	51.6 (11.2)	50.6 (11.5)	0.007
**Gender**, *n* (%)			
Female	1855 (100.0)	2375 (100.0)	
Male	0 (0.0)	0 (0.0)	
**Race**, *n* (%)			
White	1454 (78.4)	1693 (71.3)	<0.001
Black or African American	210 (11.3)	293 (12.3)	0.311
American Indian or Alaskan Native	10 (0.5)	10 (0.4)	0.579
Native Hawaiian or Pacific Islander	10 (0.5)	18 (0.8)	0.384
Asian	55 (3.0)	109 (4.6)	0.007
Other race	52 (2.8)	97 (4.1)	0.025
Unknown race	75 (4.0)	161 (6.8)	<0.001
**Ethnicity**, *n* (%)			
Not Hispanic or Latino	1699 (91.6)	1724 (72.6)	<0.001
Hispanic or Latino	98 (5.3)	156 (6.6)	0.081
Unknown ethnicity	58 (3.1)	495 (20.8)	<0.001
**Body mass index**, *n* (%)			
Underweight	66 (3.6)	69 (2.9)	0.231
Normal weight	647 (34.9)	1020 (42.9)	<0.001
Overweight	688 (37.1)	1179 (49.6)	<0.001
Obesity I	505 (27.2)	829 (34.9)	<0.001
Obesity II	279 (15.0)	461 (19.4)	<0.001
Obesity III	126 (6.8)	227 (9.6)	0.001
**Comorbidities**, *n* (%)			
Tobacco use	49 (2.6)	79 (3.3)	0.197
Diabetes	123 (6.6)	222 (9.3)	0.001
Hyperlipidemia	332 (17.9)	382 (16.1)	0.118
Hypertensive disease	557 (30.0)	744 (31.3)	0.364
Immune disorders	58 (3.1)	52 (2.2)	0.057
HIV infection	10 (0.5)	10 (0.4)	0.579
Acute and chronic kidney disease	79 (4.3)	81 (3.4)	0.151
Malignant neoplasm of the breast	1673 (90.2)	2006 (84.5)	<0.001
**Procedures**, *n* (%)			
Breast biopsy	28 (1.5)	27 (1.1)	0.288
Biologic implant placement	1509 (81.3)	1784 (75.1)	<0.001
**Medication**, *n* (%)			
Immunosuppressant	76 (4.1)	78 (3.3)	0.161
Corticosteroid	1733 (93.4)	2280 (96.0)	<0.001
**Other treatments**, *n* (%)			
Radiation	93 (5.0)	112 (4.7)	0.655
Chemotherapy	639 (34.4)	913 (38.4)	0.007

**Table 2:** Chlorhexidine cohort (*n* = 1446) and povidone cohort (*n* = 1446) characteristics after propensity score matching.

Covariate	Chlorhexidine Cohort (*n* = 1446)	Povidone Cohort (*n* = 1446)	*p*-Value
**Age**, mean (SD)	51.3 (11.4)	50.9 (11.4)	0.405
**Gender**, *n* (%)			
Female	1446 (100.0)	1446 (100.0)	
Male	0 (0.0)	0 (0.0)	
**Race**, *n* (%)			
White	1114 (77.0)	1115 (77.1)	0.965
Black or African American	164 (11.3)	154 (10.7)	0.552
American Indian or Alaskan Native	10 (0.7)	10 (0.7)	1.000
Native Hawaiian or Pacific Islander	10 (0.7)	10 (0.7)	1.000
Asian	53 (3.7)	59 (4.1)	0.563
Other race	52 (3.6)	60 (4.1)	0.441
Unknown race	54 (3.7)	50 (3.5)	0.690
**Ethnicity**, *n* (%)			
Not Hispanic or Latino	1298 (89.8)	1292 (89.3)	0.715
Hispanic or Latino	90 (6.2)	95 (6.6)	0.704
Unknown ethnicity	58 (4.0)	59 (4.1)	0.925
**Body mass index**, *n* (%)			
Underweight	48 (3.3)	50 (3.5)	0.837
Normal weight	606 (41.9)	608 (42.0)	0.940
Overweight	657 (45.4)	657 (45.4)	1.000
Obesity I	469 (32.4)	477 (33.0)	0.751
Obesity II	255 (17.6)	261 (18.0)	0.771
Obesity III	119 (8.2)	123 (8.5)	0.788
**Comorbidities**, *n* (%)			
Tobacco use	43 (3.0)	51 (3.5)	0.402
Diabetes	115 (8.0)	120 (8.3)	0.734
Hyperlipidemia	258 (17.8)	269 (18.6)	0.596
Hypertensive disease	464 (32.1)	452 (31.3)	0.631
Immune disorders	29 (2.0)	30 (2.1)	0.895
HIV infection	10 (0.7)	10 (0.7)	1.000
Acute and chronic kidney disease	56 (3.9)	58 (4.0)	0.848
Malignant neoplasm of the breast	1273 (88.0)	1269 (87.8)	0.820
**Procedures**, *n* (%)			
Breast biopsy	14 (1.0)	18 (1.2)	0.477
Biologic implant placement	1155 (79.9)	1171 (81.0)	0.453
**Medication**, *n* (%)			
Immunosuppressant	51 (3.5)	56 (3.9)	0.622
Corticosteroid	1392 (96.3)	1380 (95.4)	0.263
**Other treatments**, *n* (%)			
Radiation	76 (5.3)	76 (5.3)	1.000
Chemotherapy	522 (36.1)	526 (36.4)	0.877

**Table 3:** Risk analysis of 30-, 60-, and 90-day post-surgical outcomes comparing chlorhexidine vs. iodine povidone for skin antisepsis in TE-based breast reconstruction.

Time/Outcome	Exposure (Chlorhexidine), *n*	Control (Povidone), *n*	Risk Ratio	95% CI	*p*-Value
***30 days***					
Surgical site infection	13	21	0.62	0.31–1.23	0.168
Wound dehiscence	10	10	1.00	0.42–2.40	1.000
Emergency department visit	87	92	0.95	0.71–1.26	0.700
Debridement	17	24	0.71	0.38–1.31	0.271
TE removal	51	61	0.84	0.58–1.20	0.335
***60 days***					
Surgical site infection	29	35	0.83	0.51–1.35	0.448
Wound dehiscence	10	11	0.91	0.39–2.13	0.827
Emergency department visit	117	132	0.89	0.70–1.12	0.320
Debridement	29	34	0.85	0.52–1.39	0.524
TE removal	109	125	0.87	0.68–1.12	0.275
***90 days***					
Surgical site infection	32	43	0.74	0.47–1.17	0.198
Wound dehiscence	10	13	0.77	0.34–1.75	0.530
Emergency department visit	137	158	0.87	0.70–1.08	0.197
Debridement	36	40	0.90	0.58–1.40	0.642
TE removal	155	167	0.93	0.76–1.14	0.478

## Data Availability

Restrictions apply to the availability of these data. Data were obtained from TriNetX and are available from the authors with the permission of TriNetX.

## Abbreviations

The following abbreviations are used in this manuscript:

TE	Tissue expander
SSI	Surgical site infection
CPT	Current procedural terminology
ICD	International classification of diseases
CI	Confidence interval
RR	Risk ratio
OR	Odds ratio

## Appendix A

**Table A1:** Codes utilized for query building and outcomes.

Procedure/Diagnosis	Code
**Chlorhexidine Cohort**	
Must have	
Tissue expander-based breast reconstruction	CPT: 19357
AND (*same day*)	
Chlorhexidine	RxNorm: 2358
AND cannot have (*same day*)	
Povidone-iodine	RxNorm: 8611
**Povidone cohort**	
Must have	
Tissue expander-based breast reconstruction	CPT: 19357
AND (*same day*)	
Povidone-iodine	RxNorm: 8611
AND cannot have (*same day*)	
Chlorhexidine	RxNorm: 2358
**Outcomes**	
Surgical site infection	ICD10: T81.41XA, T81.42XA, T81.43XA, T81.49XA
Wound dehiscence	ICD10: T81.30XA
Emergency department visit	CPT: 1013711
Debridement	CPT: 1003164
Tissue expander removal	CPT: 11971, ICD10: 0HPU0NZ, 0HPT0NZ, 0HPU3NZ, 0HPT3NZ,

## References

[1] Viola G.M., Selber J.C., Crosby M., Raad I.I., Butler C.E., Villa M.T., Kronowitz S.J., Clemens M.W., Garvey P., Yang W. (2016). Salvaging the Infected Breast Tissue Expander: A Standardized Multidisciplinary Approach. Plast. Reconstr. Surg. -Glob. Open.

[2] Kraenzlin F.S., Saunders H., Aliu O., Cooney D., Rosson G.D., Sacks J.M., Broderick K., Manahan M.A. (2019). Classification of breast tissue expander infections: Back to the basics. J. Surg. Oncol..

[3] Cheadle W.G. (2006). Risk Factors for Surgical Site Infection. Surg. Infect..

[4] Spruce L. (2020). Reducing the Risk of Surgical Site Infection with Effective Preoperative Patient Skin Antisepsis. AORN J..

[5] Boyce J.M. (2023). Best products for skin antisepsis. Am. J. Infect. Control.

[6] Łukomska-Szymańska M., Barbara J. (2017). Chlorhexidine–mechanism of action and its application to dentistry. J. Stomatol..

[7] Berríos-Torres S.I., Umscheid C.A., Bratzler D.W., Leas B., Stone E.C., Kelz R.R., Reinke C.E., Morgan S., Solomkin J.S., Mazuski J.E. (2017). Centers for Disease Control and Prevention Guideline for the Prevention of Surgical Site Infection, 2017. JAMA Surg..

[8] World Health Organization (2016). Global Guidelines for the Prevention of Surgical Site Infection.

[9] Lepelletier D., Maillard J.Y., Pozzetto B., Simon A. (2020). Povidone Iodine: Properties, Mechanisms of Action, and Role in Infection Control and *Staphylococcus aureus* Decolonization. Antimicrob. Agents Chemother..

[10] Bigliardi P.L., Alsagoff S.A.L., El-Kafrawi H.Y., Pyon J.-K., Wa C.T.C., Villa M.A. (2017). Povidone iodine in wound healing: A review of current concepts and practices. Int. J. Surg..

[11] Mimoz O., Lucet J.-C., Kerforne T., Pascal J., Souweine B., Goudet V., Mercat A., Bouadma L., Lasocki S., Alfandari S. (2015). Skin antisepsis with chlorhexidine–alcohol versus povidone iodine–alcohol, with and without skin scrubbing, for prevention of intravascular-catheter-related infection (CLEAN): An open-label, multicentre, randomised, controlled, two-by-two factorial trial. Lancet.

[12] Darouiche R.O., Wall M.J.J., Itani K.M., Otterson M.F., Webb A.L., Carrick M.M., Miller H.J., Awad S.S., Crosby C.T., Mosier M.C. (2010). Chlorhexidine–Alcohol versus Povidone–Iodine for Surgical-Site Antisepsis. N. Engl. J. Med..

[13] Widmer A.F., Atkinson A., Kuster S.P., Wolfensberger A., Klimke S., Sommerstein R., Eckstein F.S., Schoenhoff F., Beldi G., Gutschow C.A. (2024). Povidone Iodine vs Chlorhexidine Gluconate in Alcohol for Preoperative Skin Antisepsis. JAMA.

[14] Zhang Z., Kim H.J., Lonjon G., Zhu Y. (2019). Balance diagnostics after propensity score matching. Ann. Transl. Med..

[15] Nguyen T.-L., Collins G.S., Spence J., Daurès J.-P., Devereaux P.J., Landais P., Le Manach Y. (2017). Double-adjustment in propensity score matching analysis: Choosing a threshold for considering residual imbalance. BMC Med. Res. Methodol..

[16] Levin I., Amer-Alshiek J., Avni A., Lessing J.B., Satel A., Almog B. (2011). Chlorhexidine and Alcohol Versus Povidone-Iodine for Antisepsis in Gynecological Surgery. J. Women’s Health.

[17] Cruz M.R.B., Amaral D.C., Gonçalves O.R., Cyrino L.G., Nascimento L.M., Barroso F.V.C., Louzada R.N., Rassi T.N.d.O., Mora-Paez D.J., Guedes J. (2025). Chlorhexidine Compared with Povidone–Iodine in Intravitreal Injection: A Systematic Review and Meta-Analysis. J. Ocul. Pharmacol. Ther..

[18] Hsieh H.-H., Yu Y., Chang C.-J., Chang T.-Y. (2025). A comparative meta-analysis of povidone–iodine–alcohol vs. chlorhexidine–alcohol for preoperative skin antisepsis in abdominal surgery. Am. J. Surg..

[19] Machinski E., da Cruz V.F., Conde R.A., Filho A.R.O., Varone B.B., Gobbi R.G., Helito C.P., Leal D.P. (2025). Chlorhexidine or Povidone-Iodine Solution Irrigation Versus Saline Irrigation for the Prevention of Postoperative Infections in Primary Total Joint Arthroplasty: A Systematic Review and Meta-Analysis. J. Arthroplast..

[20] Ademuyiwa A.O., Hardy P., Runigamugabo E., Sodonougbo P., Behanzin H., Kangni S., Agboton G., Adagrah L.A., Adjei-Acquah E., Acquah A.O. (2021). Reducing surgical site infections in low-income and middle-income countries (FALCON): A pragmatic, multicentre, stratified, randomised controlled trial. Lancet.

[21] Dior U.P., Kathurusinghe S., Cheng C., Reddington C., Daley A.J., Ang C., Healey M. (2020). Effect of Surgical Skin Antisepsis on Surgical Site Infections in Patients Undergoing Gynecological Laparoscopic Surgery. JAMA Surg..

[22] Qian J., Lin J., Liu J., Gong Y., Zheng S., Mei L., Tang X., Xie L., Li H., Zhang C. (2025). Chlorhexidine gluconate versus povidone-iodine for nasal bacteria decolonization before transsphenoidal surgery in patients with pituitary neuroendocrine tumors: A prospective, randomized, double-blind, noninferiority trial. Int. J. Surg..

[23] Charehbili A., Swijnenburg R.-J., van de Velde C., Bremer J.v.D., van Gijn W. (2014). A Retrospective Analysis of Surgical Site Infections after Chlorhexidine–Alcohol versus Iodine–Alcohol for Pre-Operative Antisepsis. Surg. Infect..

[24] Noorani A., Rabey N., Walsh S.R., Davies R.J. (2010). Systematic review and meta-analysis of preoperative antisepsis with chlorhexidine *versus* povidone–iodine in clean-contaminated surgery. Br. J. Surg..

[25] Chen S., Chen J.W., Guo B., Xu C.C. (2020). Preoperative Antisepsis with Chlorhexidine Versus Povidone-Iodine for the Prevention of Surgical Site Infection: A Systematic Review and Meta-analysis. World J. Surg..

[26] Privitera G.P., Costa A.L., Brusaferro S., Chirletti P., Crosasso P., Massimetti G., Nespoli A., Petrosillo N., Pittiruti M., Scoppettuolo G. (2017). Skin antisepsis with chlorhexidine versus iodine for the prevention of surgical site infection: A systematic review and meta-analysis. Am. J. Infect. Control.

[27] Guenezan J., Marjanovic N., Drugeon B., O Neill R., Liuu E., Roblot F., Palazzo P., Bironneau V., Prevost F., Paul J. (2021). Chlorhexidine plus alcohol versus povidone iodine plus alcohol, combined or not with innovative devices, for prevention of short-term peripheral venous catheter infection and failure (CLEAN 3 study): An investigator-initiated, open-label, single centre, randomised-controlled, two-by-two factorial trial. Lancet Infect. Dis..

[28] Wade R.G., Bourke G., Wormald J.C.R., Totty J.P., Stanley G.H.M., Lewandowski A., Rakhra S.S., Gardiner M.D. (2021). Chlorhexidine *versus* povidone–iodine skin antisepsis before upper limb surgery (CIPHUR): An international multicentre prospective cohort study. BJS Open.

[29] Beber S.A.B., Sanborn R.M.B., Miller P.E., Kasser J.R., Waters P.M., Watkins C.J., Shore B.J.M. (2022). Jumping on the Bandwagon: Comparing the Efficacy of Chlorhexidine Versus Povidone-Iodine Preoperative Skin Antiseptic in Preventing Surgical Site Infections Following Pediatric Orthopaedic Surgery. J. Pediatr. Orthop..

[30] Dörfel D., Maiwald M., Daeschlein G., Müller G., Hudek R., Assadian O., Kampf G., Kohlmann T., Harnoss J.C., Kramer A. (2021). Comparison of the antimicrobial efficacy of povidone-iodine-alcohol versus chlorhexidine-alcohol for surgical skin preparation on the aerobic and anaerobic skin flora of the shoulder region. Antimicrob. Resist. Infect. Control.

[31] Mangram A.J., Horan T.C., Pearson M.L., Silver L.C., Jarvis W.R. (1999). The Hospital Infection Control Practices Advisory Committee Guideline for Prevention of Surgical Site Infection, 1999. Infect. Control Hosp. Epidemiol..

[32] Eslami A.R.D. (2013). Antimicrobial Assay of Chlorhexidine-Wetted Textile Napkins for Surgical Site Disinfection in Ocular Surgery. Int. J. Clin. Med..

[33] Kataria K., Bagdia A., Srivastava A. (2015). Are Breast Surgical Operations Clean or Clean Contaminated?. Indian J. Surg..

[34] Oleck N.C., Gu C., Phillips B.T.M. (2022). Defining Mastectomy Skin Flap Necrosis: A Systematic Review of the Literature and a Call for Standardization. Plast. Reconstr. Surg..

[35] Bolton L. (2021). Surgical Site Infection in Cancer Patients. Wounds A Compend. Clin. Res. Pract..

[36] Calderwood M.S., Anderson D.J., Bratzler D.W., Dellinger E.P., Garcia-Houchins S., Maragakis L.L., Nyquist A.C., Perkins K.M., Preas M.A., Saiman L. (2023). Strategies to prevent surgical site infections in acute-care hospitals: 2022 Update. Infect. Control Hosp. Epidemiol..

[37] Chin K., Wärnberg F., Kovacs A., Bagge R.O. (2023). Impact of Surgical Care Bundle on Surgical Site Infection after Non-Reconstructive Breast Cancer Surgery: A Single-Centre Retrospective Comparative Cohort Study. Cancers.

[38] Campbell M.M., Turi J., Collier S., English C., Sistla V., Smith M.J., Moorthy G., Seidelman J., Smith B.A., Lewis S.S. (2025). Implementation of Bundled Interventions to Reduce Surgical Site Infections in Pediatric Patients Undergoing Cardiothoracic Surgery: A Quality Improvement Project. AORN J..

[39] Hekman K.E., Michel E., Blay E., Helenowski I.B., Hoel A.W. (2019). Evidence-Based Bundled Quality Improvement Intervention for Reducing Surgical Site Infection in Lower Extremity Vascular Bypass Procedures. J. Am. Coll. Surg..

[40] Guo X.M., Runge M., Miller D., Aaby D., Milad M. (2020). A bundled intervention lowers surgical site infection in hysterectomy for benign and malignant indications. Int. J. Gynecol. Obstet..

[41] Arroyo-Garcia N., Badia J.M., Vázquez A., Pera M., Parés D., Limón E., Almendral A., Piriz M., Díez C., Fraccalvieri D. (2022). An interventional nationwide surveillance program lowers postoperative infection rates in elective colorectal surgery. A cohort study (2008–2019). Int. J. Surg..

[42] Bogdan R.-G., Helgiu A., Cimpean A.-M., Ichim C., Todor S.B., Iliescu-Glaja M., Bodea I.C., Crainiceanu Z.P. (2024). Assessing Fat Grafting in Breast Surgery: A Narrative Review of Evaluation Techniques. J. Clin. Med..

